# Molecularly Imprinted Polymer Nanospheres with Hydrophilic Shells for Efficient Molecular Recognition of Heterocyclic Aromatic Amines in Aqueous Solution

**DOI:** 10.3390/molecules28052052

**Published:** 2023-02-22

**Authors:** Peijian Sun, Yipeng Wang, Song Yang, Xuehui Sun, Bin Peng, Lining Pan, Yunzhen Jia, Xiaobing Zhang, Cong Nie

**Affiliations:** Key Laboratory of Tobacco Chemistry, Zhengzhou Tobacco Research Institute of CNTC (China National Tobacco Corporation), No. 2 Fengyang Street, Zhengzhou 450001, China

**Keywords:** core-shell, molecularly imprinted polymers, hydrophilic shells, heterocyclic aromatic amines

## Abstract

Heterocyclic aromatic amine molecularly imprinted polymer nanospheres with surface-bound dithioester groups (haa-MIP) were firstly synthesized via reversible addition-fragmentation chain transfer (RAFT) precipitation polymerization. Then, a series of core-shell structural heterocyclic aromatic amine molecularly imprinted polymer nanospheres with hydrophilic shells (MIP-HSs) were subsequently prepared by grafting the hydrophilic shells on the surface of haa-MIP via on-particle RAFT polymerization of 2-hydroxyethyl methacrylate (HEMA), itaconic acid (IA), and diethylaminoethyl methacrylate (DEAEMA). The haa-MIP nanospheres showed high affinity and specific recognition toward harmine and its structural analogs in organic solution of acetonitrile, but lost the specific binding ability in aqueous solution. However, after the grafting of the hydrophilic shells on the haa-MIP particles, the surface hydrophilicity and water dispersion stability of the polymer particles of MIP-HSs greatly improved. The binding of harmine by MIP-HSs with hydrophilic shells in aqueous solutions is about two times higher than that of NIP-HSs, showing an efficient molecular recognition of heterocyclic aromatic amines in aqueous solution. The effect of hydrophilic shell structure on the molecular recognition property of MIP-HSs was further compared. MIP-PIA with carboxyl groups containing hydrophilic shells showed the highest selective molecular recognition ability to heterocyclic aromatic amines in aqueous solution.

## 1. Introduction

Molecularly imprinted polymers (MIPs) are artificial receptors with permanent template cavities (imprinted binding sites) in the network, which are complementary to the shape, size, and functionality of the template [1,2]. Due to the high, specifically molecular recognition toward the target analytes, as well as the easy preparation and low cost, MIPs have been widely utilized as molecular recognition and separation materials in various fields, such as chemical sensors, chromatography, sample pretreatment, and drug delivery [3,4,5,6,7,8,9,10]. MIPs can be prepared by varied polymerization techniques, among which precipitation polymerization is the most-used method. Reversible addition-fragmentation chain transfer (RAFT) polymerization has drawn great interest in MIPs preparation because it is not only compatible with a wide range of functional monomers, but also allows precise control of the composition, network structure, and end group functionality of the obtained MIPs [11]. The important advantage of RAFT polymerization utilized in MIPs preparation is that the “living” end groups can cover the surface of the MIPs and can be directly utilized for straightforward surface functionalization.

There have been many works focused on the MIPs-based solid-phase extraction of target analytes in real matrices [12,13,14,15,16,17]. However, the presently developed MIPs are normally only compatible with organic solvents, but mostly failed to show specifically molecular recognition in aqueous solutions, significantly limiting their practical application. The direct use of MIPs in real water-based matrices is more difficult and challenging because of the weakening of the imprint interaction between the template and the imprinted binding sites, and the increasing of the non-specific interaction due to the normally hydrophobic surface character of MIPs. In the past 20 years, many efforts have been devoted to developing MIPs that can be directly used for molecular recognition in aqueous solutions [18,19,20,21,22]. One important strategy is improving the surface hydrophilicity of the MIPs. For example, Zhang and coworkers [23,24,25] have prepared a series of water-compatible MIPs microspheres by grafting hydrophilic polymer shells onto the MIP microspheres, and they demonstrated that the molecular recognition ability of MIPs could be greatly improved in aqueous solutions. However, there are few studies focusing on the outer shell structure that influences the molecular recognition in aqueous solutions.

Heterocyclic aromatic amines (HAAs), which are usually formed by a complex Maillard reaction and/or thermal pyrolysis from amino acids, proteins, reducing sugars, and other precursors, are highly concerned toxicants widely found in fried food, coffee, and cigarette smoke [26,27,28]. Many sample pretreatment methods, such as solvent extraction, solid phase extraction, and solid phase microextraction, have been used for the purification of HAAs to eliminate compounds that might interfere with the analysis before the qualitative and quantitative analysis of HAAs [28,29,30,31]. However, these methods all have some drawbacks, which include low selectivity, or tedious operation.

In this study, HAA molecularly imprinted polymer nanospheres with surface-bound dithioester groups (haa-MIPs) were firstly synthesized via RAFT precipitation polymerization. Subsequently, surface grafting of hydrophilic poly(2-hydroxyethyl methacrylate) (PHEMA), poly (Itaconic acid) (PIA), and poly(diethylaminoethyl methacrylate) (PDEAEMA) outer shells was performed via on-particle RAFT polymerization, using the “living” character of the haa-MIP to construct a series of core-shell structural HAA molecularly imprinted polymer nanospheres with hydrophilic shells (MIP-HSs). The water dispersion stability and molecular recognition of HAAs by haa-MIP and MIP-HSs nanospheres were studied by equilibrium binding analysis to demonstrate the efficient HAA molecular recognition in aqueous solution.

## 2. Results and Discussion

### 2.1. Preparation and Characterization of Haa-MIP and MIP-HSs

**Figure 1:**
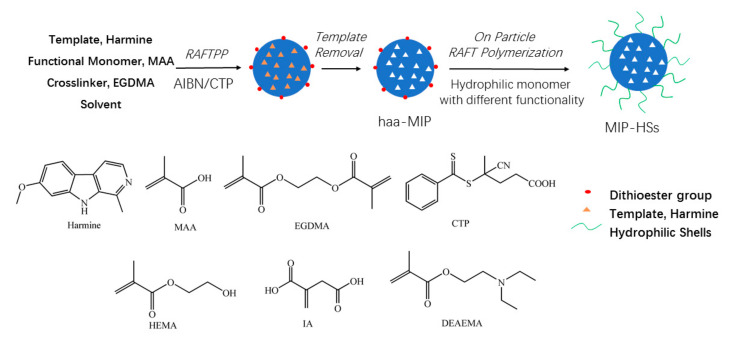
Synthesis pathway of the haa-MIP and the core-shell structural MIP-HSs.

Well-defined HAA molecularly imprinted polymer nanospheres with surface-bound dithioester groups (haa-MIPs) were firstly synthesized by RAFT precipitation polymerization of methacrylic acid (MAA) and ethylene glycol dimethacrylate (EGDMA) with harmine as template. Then, using the haa-MIP with “living” dithioester end groups as the immobilized RAFT agent, on-particle RAFT polymerization of hydrophilic monomers, such as 2-hydroxyethyl methacrylate (HEMA), itaconic acid (IA), and diethylaminoethyl methacrylate (DEAEMA), resulted in a series of core-shell structural HAA molecularly imprinted polymer nanospheres with hydrophilic shells (MIP-HSs) (Figure 1).

**Figure 2:**
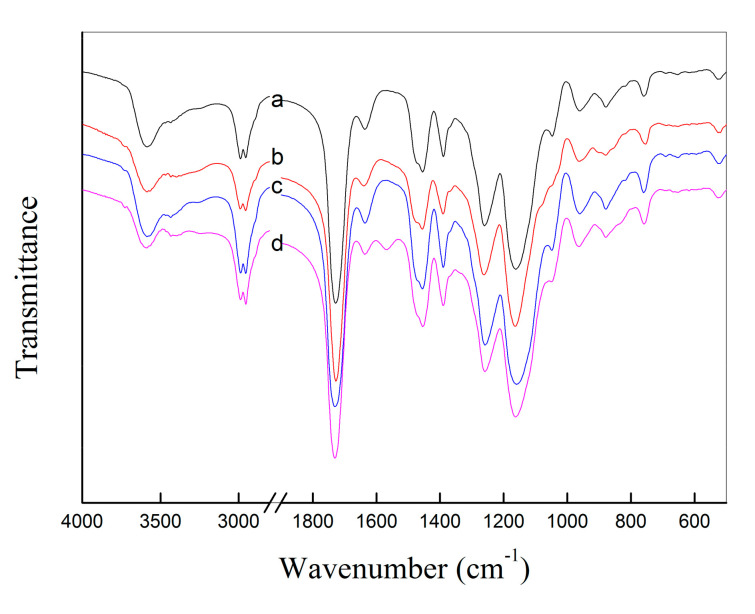
FTIR spectra of (**a**) haa-MIP, (**b**) MIP-PHEMA, (**c**) MIP-PIA, (**d**) MIP-PDEAEMA.

The obtained un-grafted haa-MIP and grafted MIP-HSs nanospheres were characterized by Fourier transform infrared (FTIR) spectra. As seen in Figure 2, the significant peaks around 1730 cm^−1^ (C=O stretching vibration), 1260 cm^−1^, and 1160 cm^−1^ (C−O−C stretching vibration) belonged to the ester groups in the EGDMA units, which verified the existence of EGDMA units. Meanwhile, the peaks at around 3580 cm^−1^ (O-H stretching vibration), as well as the 1730 cm^−1^ peak (C=O stretching vibration), confirmed the existence of MAA units in both the un-grafted haa-MIP and the grafted MIP-HSs particles. The small peak around 1630 cm^−1^ in the FTIR spectra is ascribed to the C=C stretching vibration, which is from the un-crosslinked EGDMA units in the MIPs. The characteristic band appeared at around 1565 cm^−1^ (C–N stretching vibration band) in the FTIR spectrum of MIP-PDEAEMA, indicating the successful surface grafting of PDEAEMA shells onto the precursor of haa-MIP beads. It should be noted that the characteristic absorption peaks of PHEMA and PIA chains were overlapped by the haa-MIP core in the FTIR spectra of MIP-PHEMA and MIP-PIA.

**Figure 3:**
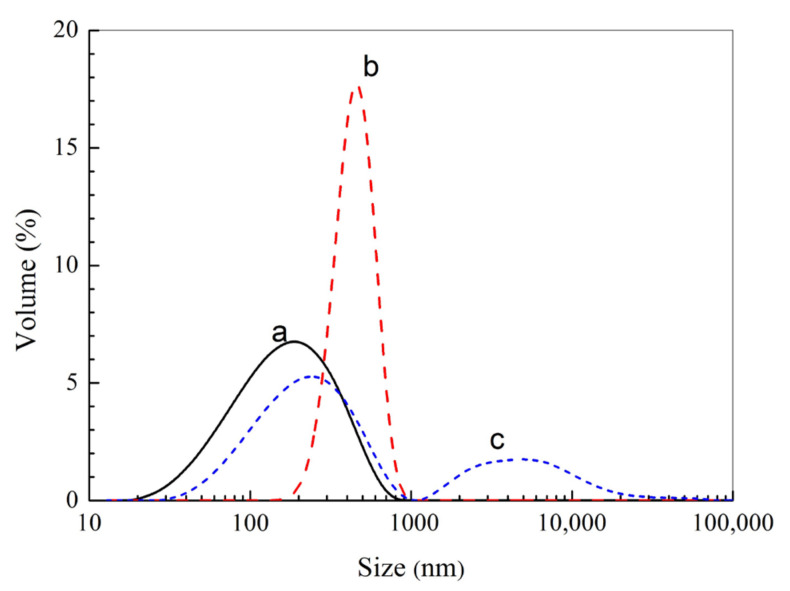
Size distribution of haa-MIP and NIP. (**a**) haa-MIP in acetonitrile. (**b**) NIP in acetonitrile. (**c**) haa-MIP in water.

**Figure 4:**
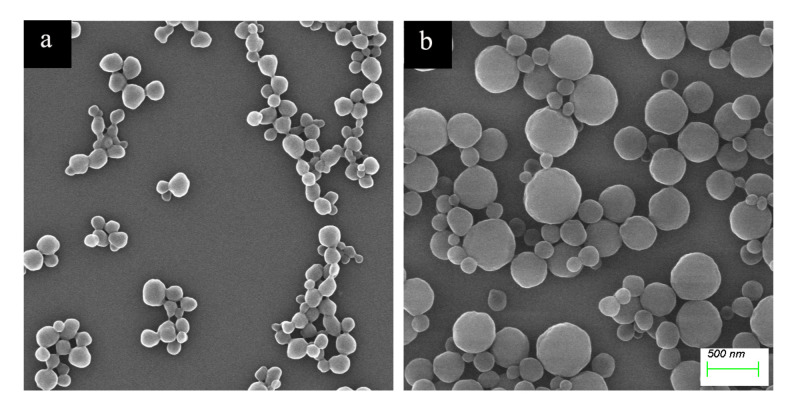
SEM photographs of (**a**) haa-MIP and (**b**) NIP nanospheres.

Figure 3 shows the particle size distribution of the haa-MIP and the non-imprinted polymer (NIP) nanospheres. The average particle size of the haa-MIP and NIP in acetonitrile is about 190 nm and 460 nm, respectively (Figure 3). The particle size of haa-MIP is obviously lower than that of NIP. The difference of particle size between the haa-MIP and NIP particles could be further confirmed by SEM observation in Figure 4. A size distribution in the range of 60–220 nm is observed for haa-MIP, while the NIP has a relatively broad size distribution in the range of 90–550 nm. The average sizes of haa-MIP and NIP are about 145 nm and 290 nm, as determined from the SEM images. The size difference between the haa-MIP and NIP nanospheres is closely related to the imprinting reaction during the preparation of MIP nanospheres, which is in consistent with the previous studies [5,24]. Notably, when the haa-MIP nanospheres were dispersed in water, an aggregation peak at about 5000 nm was shown in the size distribution of haa-MIP (Figure 3c), which is probably due to the hydrophobic surface characteristic.

**Figure 5:**
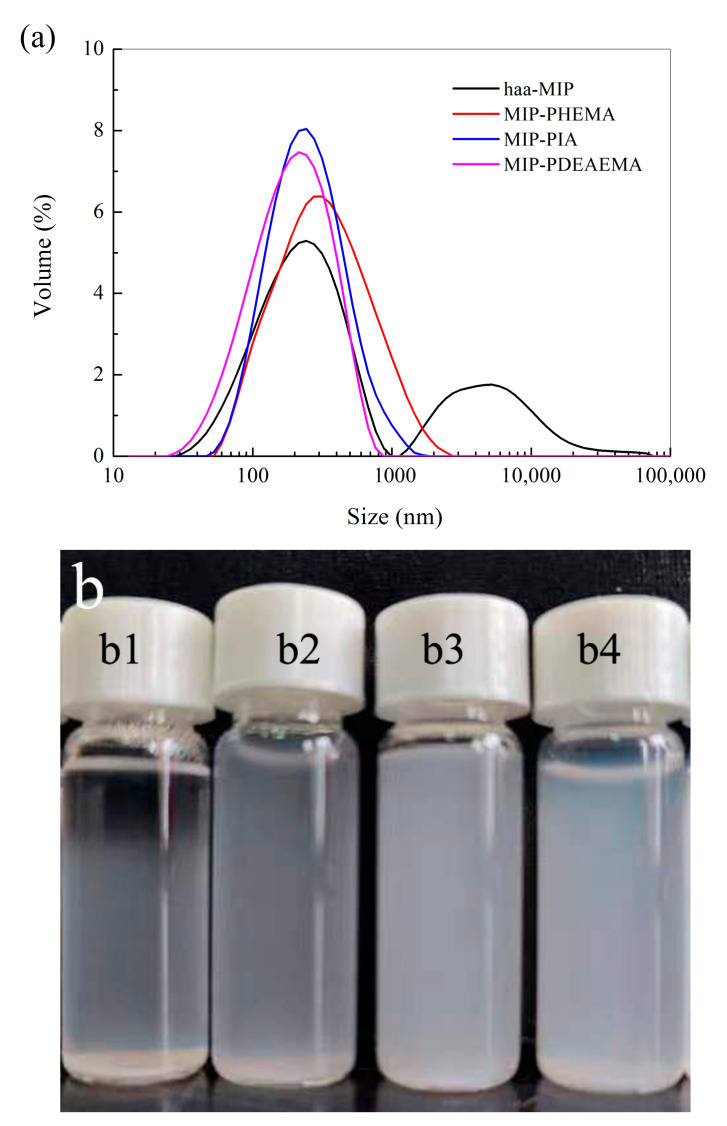
(**a**) Size distribution of haa-MIP and MIP-HSs in water. (**b**) The dispersion photographs of haa-MIP and MIP-HSs in pure water after settling down for 1 h at 25 °C. The samples of b1–b4 are haa-MIP, MIP-PHEMA, MIP-PIA, and MIP-PDEAEMA, respectively.

To improve the surface hydrophilicity of MIPs beads, grafting of hydrophilic shells was achieved by on-particle RAFT polymerization of hydrophilic monomers. The resultant core-shell structural MIP-HSs were then characterized by laser diffraction particle size analyzer, and the results are shown in Figure 5a. After the grafting of hydrophilic shells on the haa-MIP nanospheres, no obvious aggregation peak was found in the size distribution of the core-shell structural MIP-HSs in aqueous solution, indicating that the grafting of hydrophilic shells could greatly improve the water-compatibility of MIP-HSs. The average particle sizes of MIP-PHEMA, MIP-PIA, and MIP-PDEAEMA in aqueous solution are 280 nm, 240 nm, and 210 nm, respectively, slightly larger than haa-NIP. These results could also indicate the successful grafting of hydrophilic shells on the haa-MIP particles and demonstrate that the introduction of hydrophilic shells on the MIPs particles is beneficial to inhibiting the tendency of aggregation.

Figure 5b shows the dispersion photographs of haa-MIP and MIP-HSs in pure water after settling down for 1 h. The dispersion of haa-MIP showed an obvious stratification after settling down; the upper solution is relatively clear, and the bottom of the bottle has nanosphere aggregate settling. However, the dispersions of MIP-HSs, such as MIP-PHEMA, MIP-PIA, and MIP-PDEAEMA, were still nearly uniformly dispersed after settling down, indicating that the introduction of hydrophilic shells of PHEMA, PIA, and PDEAEMA could greatly improve the water dispersibility of MIP-HSs due to the improved surface hydrophilicity. These results are consistent with the particle size distribution results characterized by laser diffraction. Notably, it is found that MIP-PIA and MIP-PDEAEMA have better water dispersibility than MIP-HEMA, when comparing the three kinds of MIP-HSs. The better water dispersibility of MIP-PIA and MIP-PDEAEMA probably resulted from the carboxyl and/or amino groups in the outer shells, which could improve the water dispersibility of MIP-PIA and MIP-PDEAEMA with the electrostatic effect of the functional groups.

**Figure 6:**
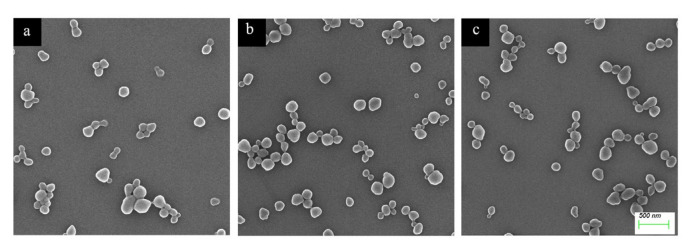
SEM photographs of (**a**) MIP-PHEMA, (**b**) MIP-PIA, and (**c**) MIP-PDEAEMA.

The morphology of MIP-HSs was observed by SEM. As shown in Figure 6, MIP-PHEMA, MIP-PIA, and MIP-PDEAEMA all have relatively regular spherical morphology, and their average particle sizes are about 180 nm. MIP-HSs have similar spherical structures, but slightly larger sizes as compared to the precursor of haa-MIP. The larger size and improved water dispersibility could also indicate the successful grafting of hydrophilic shells.

### 2.2. Molecular Recognition Property of Haa-MIP Particles

**Figure 7:**
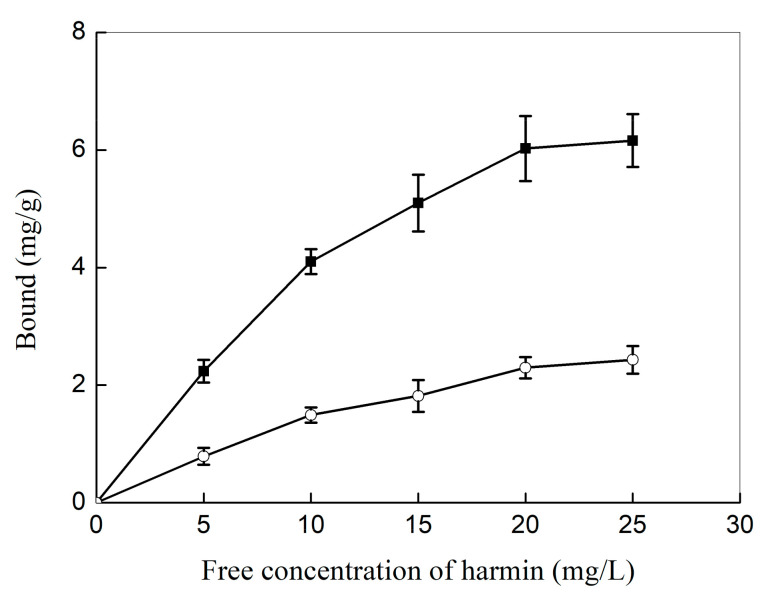
Equilibrium binding of haa-MIP (square) and NIP (circular) nanospheres toward harmine in acetonitrile.

To verify the molecular selectivity of the haa-MIP nanospheres toward HAA, equilibrium binding of the haa-MIP and the NIP nanospheres toward the template of harmine in acetonitrile were firstly compared, and the results are shown in Figure 7. The uptake of harmine by the haa-MIP is 2–3 times higher than that of the NIP. For instance, the haa-MIP could uptake 6.03 mg/g of harmine, while the uptake by NIP is only about 2.30 mg/g at the initial concentration of 20 mg/L harmine in acetonitrile. These results indicated that the haa-MIP had a significantly higher affinity and specific recognition toward harmine due to the molecularly imprinted sites in its network. In addition, the smaller size of haa-MIP may also have a positive contribution to the high binding capacity. The specific harmine-binding ability of haa-MIP is supposed to be mediated by the imprint interaction between the basic amine moiety in harmine and the molecularly imprinted sites in haa-MIP beads.

**Figure 8:**
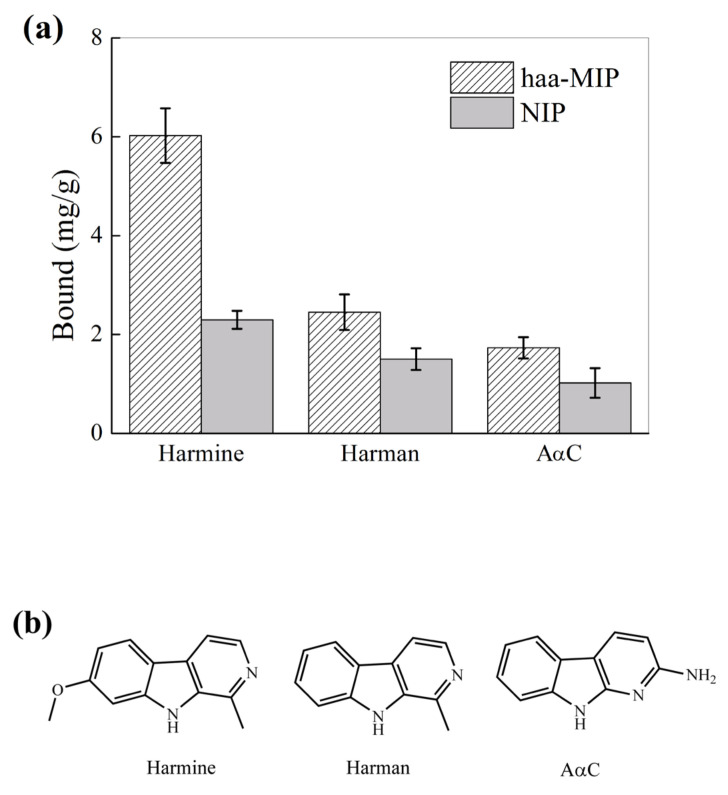
(**a**) Equilibrium binding of haa-MIP and NIP nanospheres toward harmine and its structural analogues (20 mg/L) in acetonitrile. (**b**) Chemical structure of harmine and its structural analogues.

To further study the selectivity of haa-MIP nanospheres toward other HAAs that are structural analogs of harmine, the binding property of haa-MIP and NIP nanospheres toward harman and AαC was further investigated, which is shown in Figure 8. The binding of haa-MIP toward harmine, harman, and AαC was 6.0, 2.5, and 1.7 mg/g, respectively, which is 2.5, 1.7, and 1.7 times higher, respectively, than the control NIP. The results further confirmed that it is the imprinted sites, but the random carboxyl groups that resulted in the high harmine affinity and specific recognition of haa-MIP, and they demonstrated that the haa-MIP showed enhanced specific binding ability to HAAs that have analogous structure to the template of harmine. To our knowledge, there have been only a few reports on MIPs for HAAs. Chen and co-workers [32] have reported magnetic molecularly imprinted polymers (MMIP) for HAAs. The adsorptive capacity of MMIPs toward 2-Amino-3-methylimidazo [4,5-f]quinoline (IQ, the template used) and harman (structural analog to IQ) in acetonitrile was 2.07 mg/g and 0.015 mg/g. The present reported haa-MIP shows higher binding capacities toward HAAs, with a comparative imprinting factor as compared with the previous report [32].

**Figure 9:**
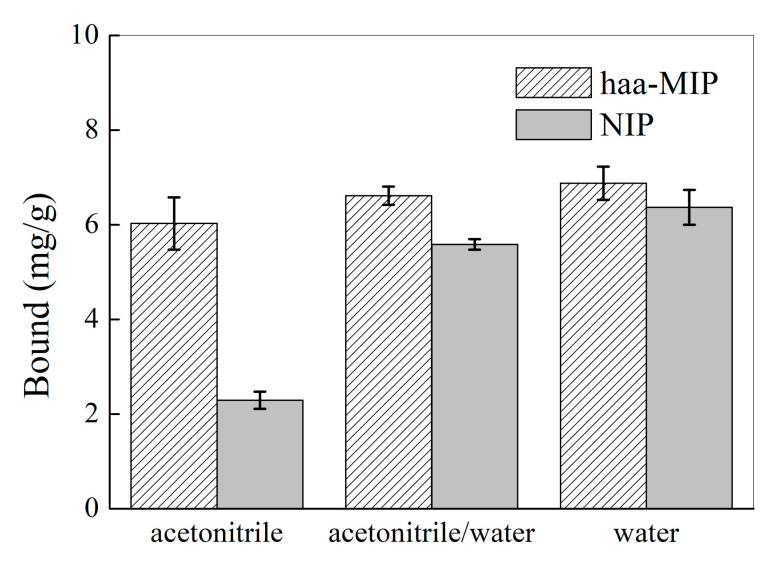
Equilibrium binding of the haa-MIP and NIP nanospheres toward harmine (20 mg/L) in acetonitrile, acetonitrile/water (*v*/*v* 50/50), and water.

It has been well demonstrated that the solvent, such as water, could play an important role in the MIPs binding process because the solvent can not only affect the MIPs’ surface properties, but also can have great influence on the interactions (such as hydrogen bond) between the MIPs and the target analytes. Figure 9 shows the effect of the solvent, such as water content, on the selective binding of harmine by the haa-MIP and NIP nanospheres. In acetonitrile, the uptake of harmine by haa-MIP was about 2.5 times higher than NIP. However, with water content increasing, for example, in a 50% water-contained solution and/or pure water, the uptake of harmine by haa-MIP was only slightly higher than NIP; both the haa-MIP and NIP nanospheres displayed high harmine binding due to the non-specific interactions with harmine. The non-specific binding was significantly increased upon the increase of the water content due to the hydrophobic surface properties of haa-MIP and NIP beads. The haa-MIP has the best specific binding ability toward harmine in pure acetonitrile, and the specific binding ability was nearly lost in aqueous solution. The poor selective binding of haa-MIP toward the target analytes probably resulted from the hydrophobicity surface characters of the haa-MIP and the weakening of the imprint interaction between the imprinted sites and the harmine. The non-specific interaction, for instance, hydrophobic interaction, could increase greatly in the aqueous solutions due to the hydrophobic surface character of haa-MIP, greatly inhibiting the specific interaction. In addition, the hydrogen bond interaction between the template and the imprinted binding sites would be weakened in the aqueous solution. These results are in agreement with the previous findings [5,24,25].

### 2.3. Molecular Recognition Property of MIP-HSs Particles in Aqueous Solution

To reduce the non-specific binding and improve the specific binding of harmine in aqueous solution, hydrophilic polymer shells were grafted onto the haa-MIP to obtain core-shell structural HAA molecularly imprinted polymer nanospheres with hydrophilic shells (MIP-HSs).

**Figure 10:**
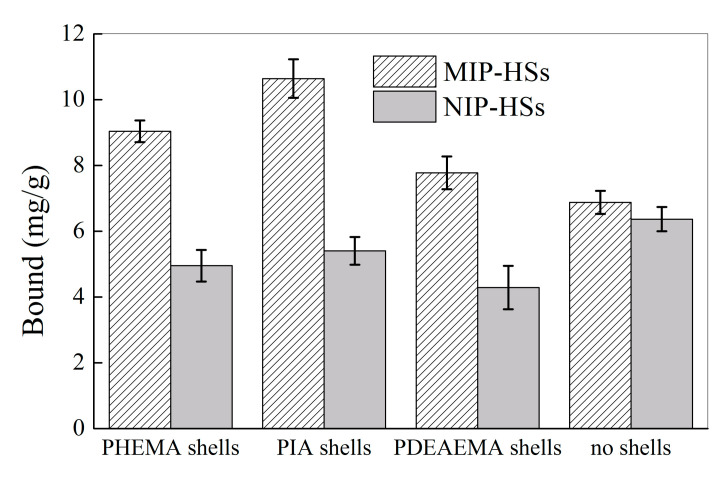
Equilibrium binding of the MIP-HSs and NIP-HSs nanospheres toward harmine (20 mg/L) in water.

Figure 10 shows the uptake of harmine by MIP-HSs with different hydrophilic shells in water. With the grafting of the hydrophilic shells, the uptake of harmine by MIP-HSs in aqueous solutions was 1.8–2.0 times higher than that of NIP-HSs. For example, MIP-PHEMA, MIP-PIA, and MIP-PDEAEMA could uptake 9.0, 10.6, and 7.8 mg/g of harmine, respectively, while only 5.0, 5.4, and 4.3 mg/g of harmine was bound to the corresponding NIP-HSs. However, both the haa-MIP and NIP, without grafting the hydrophilic shells, have a nearly equivalent bound amount of harmine in aqueous solutions. These results indicated that the grafting of hydrophilic shells could greatly improve the selective binding of MIP in aqueous solution. Compared to the NIP without hydrophilic shells, NIP-HSs with different hydrophilic shells all showed a decreased binding ability to harmine, indicating that the hydrophilic shells could greatly decrease the non-specific binding, such as the hydrophobic interaction. This is probably an important contribution to the enhanced selective binding of harmine by MIP-HSs in water. In addition, the grafting of hydrophilic shells could improve the specific binding of harmine; the hydrophilic shells benefit the template interaction with the molecularly imprinted sites in MIP-HSs. For instance, the uptake of harmine increased from 6.9 mg/g to 9.0, 10.6, and 7.8 mg/g after the grafting of hydrophilic shells of PHEMA, PIA, and PDEAEMA, respectively. The effect of hydrophilic shell structure on the molecular recognition property of MIP-HSs was compared. The results showed that the largest harmine binding amount and best specific binding ability could be achieved by the MIP-PIA nanospheres with carboxyl groups containing hydrophilic shells. The carboxyl groups in the outer shells benefit the template of harmine entering into the core of the MIP-PIA nanospheres and interacting with the imprinted sites, which resulted in the best selective molecular recognition ability.

**Figure 11:**
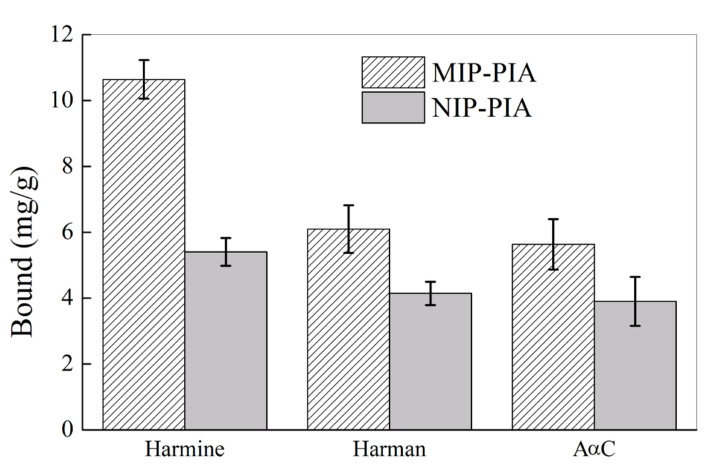
Equilibrium binding of MIP-PIA and NIP-PIA nanospheres toward harmine and its structural analogues (20 mg/L) in water.

To further study the selectivity of MIP-PIA nanospheres to HAAs that are structural analogs of harmine, the binding of MIP-PIA and NIP-PIA nanospheres to two other HAAs, harman and AαC, was further investigated. Figure 11 shows the equilibrium binding of harmine and its structural analogues to the MIP-PIA and NIP-PIA nanospheres. The binding of harman and AαC to MIP-PIA was only around 5.6–6.1 mg/g, which is significantly lower than harmine binding of 10.6 mg/g. Both harmine and its structural analogues showed a higher binding amount by MIP-PIA as compared with NIP-PIA, indicating that the MIP-PIA showed selective molecular recognition ability to HAA that has analogous structure to the template of harmine. The high selective molecular recognition ability of MIP-HSSs (e.g., MIP-PIA) toward HAAs in aqueous solution would endow it great potential in many applications, for instance, as solid phase extraction materials in HAA analysis in food and biological sample and/or as chemical sensors for HAA.

## 3. Materials and Methods

### 3.1. Materials

Methacrylic acid (MAA), Ethylene glycol dimethacrylate (EGDMA), 2-Hydroxyethyl methacrylate (HEMA), Itaconic acid (IA), diethylaminoethyl methacrylate (DEAEMA), 2,2′-azobisisobutyronitrile (AIBN), 4,4’-Azobis(4-cyanovaleric acid) (V501), harmine, harman, and 2-amino-9H-pyrido [2-3-b]indole (AαC) were purchased from Shanghai Aladdin Biochemical Technology Co., Ltd. (Shanghai, China). 4-cyano-4-(phenylcarbonothioylthio)pentanoic acid (CTP) was purchased from Sigma-Aldrich (St. Louis, MO, USA). MAA, HEMA, and DEAEMA were purified by vacuum distillation before use. AIBN and V501 were recrystallized from ethanol before use. MilliQ deionized water (Millipore system, Burlington, MA, USA) was used. Other materials were used as received.

### 3.2. Synthesis of Haa-MIP with Surface-Bound Dithioester Groups

The haa-MIP nanospheres with surface-bound dithioester groups were synthesized via RAFT precipitation polymerization by using harmine as template. Typically, MAA (0.254 mL, 3.0 mmol), harmine (212 mg, 1.0 mmol), EGDMA (2.830 mL, 15 mmol), CTP (100 mg, 0.36 mmol), AIBN (20 mg, 0.12 mmol), and acetonitrile (80 mL) were placed in a dry glass ampule equipped with a magnetic stirring bar, and then the solution was degassed under reduced pressure by triple freeze–pump–thawing cycles. After being purged with argon, the reaction mixture was sealed and stirred at 25 °C for another 2 h in order to allow the self-assembly of the functional monomer and template. Then, the polymerization was carried out at 70 °C for 16 h. The reaction was stopped by rapid cooling with liquid nitrogen, and the resulting polymer particles were obtained by centrifugation and then washed thoroughly with acetonitrile, methanol/acetic acid (9:1 *v*/*v*), and methanol successively until no template could be detected in the washing solution. After being dried at 40 °C under vacuum for 48 h, light pink haa-MIP nanospheres were obtained. The none-imprinted polymer (NIP) nanospheres were also prepared and purified as control under the identical conditions, except that no template was added to the polymerization solution.

### 3.3. Synthesis of Core-Shell Structural MIP-HSs with Hydrophilic Shells

A series of core-shell structural MIP-HSs with hydrophilic shells were prepared by on-particle RAFT polymerization of hydrophilic monomers (such as HEMA, IA, and DEAEMA) using the above obtained “living” haa-MIP nanospheres as the immobilized RAFT agent and a certain amount of sacrificial RAFT agent of CTP [5]. The typical polymerization procedure was as follows: the “living” haa-MIP nanospheres (200 mg), hydrophilic monomers (40 mmol), V501 (5 mg, 0.018 mmol), CTP (10 mg, 0.036 mmol), and methanol/water (3:1 *v*/*v*, 30 mL) were added into a dry glass ampule equipped with a magnetic stirring bar. After being degassed by three freeze–pump–thaw cycles and purged with argon, the reaction mixture was sealed and polymerization at 70 °C for 12 h. The resultant polymer particles were collected by centrifugation and then washed thoroughly with methanol/water (3:1 *v*/*v*) and methanol successively, before drying at 40 °C under vacuum. The none-imprinted polymer with different hydrophilic shells (NIP-HSs) were also prepared as control using the same polymerization procedure, except for replacing the haa-MIP nanospheres by NIP particles.

### 3.4. Characterizations

Fourier transform infrared (FTIR) spectra were measured on a Bruker Tensor 27 spectrometer by pressing the sample to be tested into a potassium boride (KBr) disk. The size distribution of the particle was determined by laser diffraction on a Mastersizer 3000 particle size analyzer. The microscopic morphology was observed by a Zeiss Gemini 300 field emission electron scanning microscope (SEM) with an accelerating voltage of 10 kV.

### 3.5. Equilibrium Binding Analysis

To 5.0 mL of solution of HAA (e.g., harmine, harman, and AαC), 5.0 mg of polymer particles (e.g., haa-MIP and/or MIP-HSs) was added. The mixture was then stirred on a rocking table at 25 °C for 12 h. Supernatant (3.0 mL) was collected from each sample by centrifugation separation, and the concentration of the un-bound HAA was subsequently measured using a Cary-100 UV-vis spectrophotometer. The bound amount of the HAA was calculated by the difference between the initial and the un-bound HAA after the binding procedure.

## 4. Conclusions

HAA molecularly imprinted polymer nanospheres with surface-bound dithioester groups (haa-MIPs) were firstly synthesized via RAFT precipitation polymerization. With the “living” haa-MIP nanospheres as the immobilized RAFT agent, a series of core-shell structural HAA molecularly imprinted polymer nanospheres with hydrophilic shells (MIP-HSs) were subsequently prepared by on-particle RAFT polymerization of hydrophilic monomer of HEMA, IA, and DEAEMA. The haa-MIP showed high affinity and specific recognition toward harmine and its structural analogs in pure acetonitrile, but nearly lost the specific binding ability in aqueous solution. After the grafting of the hydrophilic shells on haa-MIP particles, the binding of harmine by MIP-HSs in aqueous solutions is about two times higher than that of NIP-HSs, showing an improved molecular recognition in aqueous solutions. The effect of the hydrophilic shell structure on the molecular recognition property of MIP-HSs was also compared. The highest selective molecular recognition toward HAA was achieved by MIP-PIA with carboxyl groups containing hydrophilic shells. These MIP-HSs may be used in the solid phase extraction procedure for heterocyclic aromatic amines analysis in food and biological samples and/or as chemical sensor building blocks for heterocyclic aromatic amines.

## Data Availability

Not applicable.
